# An aggressive cabergoline-resistant, temozolomide-responsive macroprolactinoma due to a germline *SDHB* pathogenic variant in the absence of paraganglioma or pheochromocytoma

**DOI:** 10.3389/fendo.2023.1273093

**Published:** 2023-12-13

**Authors:** Ali S. Alzahrani, Abdulghani Bin Nafisah, Meshael Alswailem, Yosra Moria, Dagmara Poprawski, Hindi Al-Hindi, Karel Pacak

**Affiliations:** ^1^ Department of Molecular Oncology, King Faisal Specialist Hospital and Research Centre, Riyadh, Saudi Arabia; ^2^ Department of Medicine, King Faisal Specialist Hospital and Research Centre, Riyadh, Saudi Arabia; ^3^ College of Science, King Saud University, Riyadh, Saudi Arabia; ^4^ Oncology Centre, King Faisal Specialist Hospital & Research Centre, Riyadh, Saudi Arabia; ^5^ College of Medicine and Public Health, Flinders University, Adelaide, SA, Australia; ^6^ Department of Pathology and Laboratory Medicine, King Faisal Specialist Hospital and Research Centre, Riyadh, Saudi Arabia; ^7^ Section on Medical Neuroendocrinology, Eunice Kennedy Shriver National Institute of Child Health and Human Development, National Institutes of Health, Bethesda, Maryland, United States

**Keywords:** macroprolactinoma, SDHB, pituitary adenoma, cabergoline, temozolomide

## Abstract

**Context:**

Germline succinate dehydrogenase subunit B (*SDHB*) pathogenic variants are characteristic of familial paraganglioma (PGL) syndrome type 4. This syndrome frequently presents with abdominal PGL and has high tendency for locally aggressive behavior and distant metastasis. The vast majority of pituitary adenomas (PAs) are sporadic. However, PAs can be part of a number of familial tumor syndromes such as multiple endocrine neoplasia type 1 (MEN 1) or more rarely in association with pheochromocytoma and PGL (referred to as 3P syndrome). Only a limited number of PAs in association with *SDHB*-related PGL has been reported and the vast majority occurred subsequently or simultaneously with pheochromocytoma/PGL (collectively abbreviated as PPGL). In this report, we describe a young patient who had a giant pituitary macroprolactinoma resistant to large doses of cabergoline (CBG) and external beam radiotherapy (XRT). The patient did not have personal history of PPGL but was found to carry a germline *SDHB* pathogenic variant.

**Case report:**

A 38-year-old woman presented with headache, visual disturbances and galactorrhea and was found to have a 34-mm macroprolactinoma. She was treated with CBG 3-4 mg per week but PA continued to grow and caused significant cranial pressure symptoms. She underwent two transsphenoidal surgeries with rapid tumor recurrence after each one. She received XRT but PA continued to grow. She was finally treated with temozolomide with excellent response. Whole exome and subsequent Sanger sequencing confirmed that she has a pathogenic monoallelic *SDHB* mutation (NM_003000:c.C343T, p.R115*). PA tissue showed loss of heterozygosity for the same mutation and absent SDHB immunostaining confirming the pathogenic role of this *SDHB* mutation.

**Conclusion:**

Germline *SDHB* mutations can rarely cause PA in the absence of PPGL. They should be considered as a possible cause of aggressiveness and resistance to dopamine agonists in similar cases.

## Introduction

Pituitary adenomas (PAs) are common neuroendocrine tumors representing about 10-16% of intracranial tumors (1, 2). Prolactinomas are the most common functional PAs (3). They are frequently microadenomas (< 1 cm in size) but approximately 40% are macroadenomas and a small minority are giant macroprolactinomas (> 4 cm in size) (4). In contrast to almost all other PAs in which transsphenoidal surgery (TSS) is the standard therapeutic modality, prolactinomas are primarily treated medically using dopamine agonists (5, 6). However, about 10-20% of prolactinomas are resistant to dopamine agonist therapy (7). Resistant prolactinomas have been defined as those that fail to normalize prolactin level on maximal doses of dopamine agonists and/or failure of tumor shrinkage by at least 50% of the original size (7, 8). The causes of resistance in this subgroup of prolactinomas are not fully understood (7). Decreased expression of dopamine receptor type 2 has been reported (9). The dopamine agonist binding is normal and dopamine receptor pathogenic variants have been rarely described in PA (8, 10, 11). One previous study described a pathogenic variant in a case of resistant prolactinoma (10). In this report, we explore an *SDHB* pathogenic variant as a possible cause of resistance to cabergoline (CBG) in a patient with a giant prolactinoma.

Succinate dehydrogenase (SDH) is a crucial enzyme in the Krebs cycle converting succinate to fumarate and participating in the electron transfer pathway that ultimately leads to activation of the mitochondrial respiratory chain complex III (12). SDH is composed of four primary subunits, SDHA, SDHB, SDHC, and SDHD. In addition, SDHA needs an SDHA-associated factor 2 (SDHAF2) to function. SDHA and SDHB have a catalytic function while SDHC and SDHD have an anchoring function attaching the SDH subunits to the inner mitochondrial membrane. SDHAF2 catalyses the flavination of SDHA (12). Germline or somatic pathogenic variants in all of these subunits have been associated with paraganglioma (PGL) and pheochromocytoma (PCA), collectively abbreviated as PPGL (13). Occasional associations with other types of tumors have been described. These include gastrointestinal stromal tumors (14), renal cell cancer (15) and anecdotally neuroendocrine tumors (16) and lymphoid malignancy (17). PAs have been rarely described in association with PPGL (18). Some of these cases were associated with *SDHx* pathogenic variants and in some cases, genetic studies were either not performed or no genetic variant was found (18). In previous reports in which PAs was diagnosed in patients carrying germline *SDHx* pathogenic variants, PPGL either preceded or co-occurred with PAs (18). In addition, cases of PAs were diagnosed in patients with family history of PPGL either with or without confirmed *SDHx* mutations (18).

In this report, we describe a unique case of an aggressive CBG-resistant giant macroprolactinoma in a young woman who had no evidence of PPGL or any other syndromic presentations. She was found to have a previously described truncating germline *SDHB* pathogenic variant. Although her family history was initially negative for PPGL or other tumors, a 14-year old niece has recently been diagnosed with an abdominal PGL and carries the same germline *SDHB* pathogenic variant. Immunohistochemical stain showing loss of SDHB expression and loss of heterozygosity (LOH) in the tumor tissue confirmed that the *SDHB* pathogenic variant found in this patient is the underlying cause of PA and likely the reason for the aggressiveness of macroprolactinoma and resistance to CBG.

## Patients and methods

### Patient

**Figure 1:**
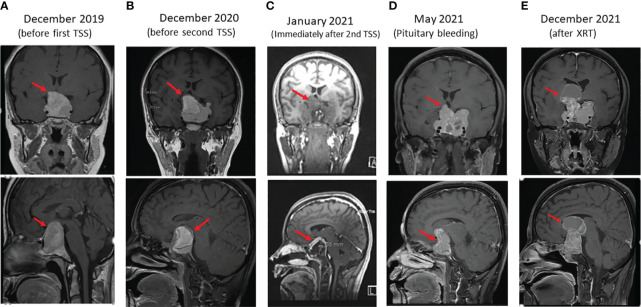
Sequential coronal (upper panels) and sagittal (lower panels) enhanced T1 weighted MR Images of macroprolactinoma at several stages (arrows) as follows: **(A)** before first trans sphenoidal surgery (TSS), **(B)** before second TSS, **(C)** immediately after Second surgery, **(D)** 4 months after second TSS presenting with bleeding in PA, and **(E)** 4 months after XRT.

A 38-year-old woman presented in 2012 with history of frontal headache, visual disturbances, intermittent amenorrhea and galactorrhea for 9 months. She was seen in an outside hospital and told to have high serum prolactin levels. Without further investigations, she was treated intermittently with CBG. Her headache and visual disturbances modestly improved and she got pregnant in 2015. During pregnancy, her headache and visual disturbances significantly worsened. She was not evaluated until 2018 when an MRI of the pituitary gland showed a 34 mm pituitary macroadenoma. Her prolactin level at that time was 573 ng/ml (normal range 3.4-24.1). She was treated with CBG 1 mg three times/week for 1.5 years. She did not improve and a repeat MRI one year later showed the size of the PA had increased to 38 mm (**Figure 1A**). For that reason, she underwent TSS in February 2020 and treated postoperatively with CBG 1 mg 4 times per week. The headache and visual disturbances continued to interfere with her daily life. No postoperative imaging was done until October 2020 when an MRI of the pituitary gland showed a large sellar and suprasellar complex lesion measuring 38 x 37 x 35 mm with significant mass effect on the adjacent brain parenchyma. She was referred to our institution in November 2020. On evaluation, she reported severe frontal headache, dizziness, decreased visual acuity, especially on the lateral gaze, menstrual irregularity, and galactorrhea. She denied changes in the size of her hands and feet or changes in facial features. Her past medical history was unremarkable except for what was mentioned above. In addition, following her first TSS, she was found to have panhypopituitarism presenting with hypothyroidism, hypoadrenalism, and hypogonadism but no history of diabetes insipidus. Therefore, she was on L-thyroxine 100 mcg daily, hydrocortisone 15 mg QAM and 5 mg QPM, and CBG 4 mg per week. Family history was negative for PAs, familial tumor syndromes or any other significant illness. In particular, no family history of PA or PPGL. However, a 14-year old paternal niece was diagnosed recently (2022) with an abdominal PGL. The patient has four children; the youngest is 4-year-old and all are well. The other family members have not yet been screened for the same *SDHB* variant. She had amenorrhea for the last 2 years prior to her presentation in 2020. She does not smoke or drink alcohol. Physical examination revealed bilateral optic nerve atrophy and bitemporal hemianopsia. No other neurological deficits were found. There were no signs of acromegaly. She was clinically euthyroid and with no clinical signs of adrenal insufficiency. The rest of her physical examination was unremarkable. Laboratory evaluation revealed prolactin 206 ng/ml (normal range 3.4-24.1) on CBG 4 mg per week, IGF-1 71 ng/ml (normal range 93-245), FT4 15.5 pmol/l (normal range 12-22) on L-thyroxine 100 mcg daily, LH 1.5 u/l (normal range 3.5-12.5), FSH 4.4 u/l (Normal range 2.4-12.6). Following withholding of hydrocortisone for 24 hr, serum cortisol levels were 81, 261 and 356 nmol/l at baseline, 30 minutes and 60 minutes after cosyntropin 250 mcg iv bolus, respectively, indicating adrenal insufficiency. An MRI of the pituitary gland revealed a 38 x 37 x 35 mm PA (**Figure 1B**).

**Figure 2:**
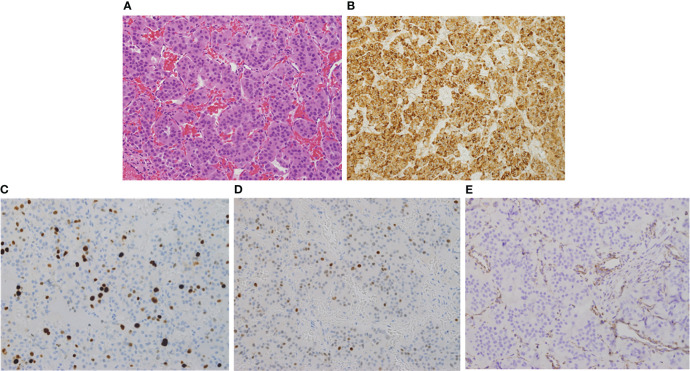
Hematoxylin and Eosin (H & E, x200) stain showing uniform polygonal tumor cells with bland nuclei and moderate eosinophilic cytoplasm **(A)**. The cells stain strongly and diffusely positive for prolactin **(B)**. The proliferation (Ki67) index is ~15% **(C)** and show increased expression of p53 **(D)**. SDHB is absent in the tumor cells while retained in endothelial and stromal cells serving as positive control **(E)**.

Due to the large compressing PA, the patient’s significant symptoms and signs and resistance to large doses of CBG, she underwent a second TSS in December 2020. Her postoperative course was uneventful. Histomorphologically, the tumor cells were arranged in nests and thick cords of polygonal cells having uniform nuclei with stippled chromatin and eosinophilic cytoplasm (**Figure 2A**). No clear cell component was noted. Although, intracytoplasic vacuoles were described as a characteristic histopathological feature in *SDHB*-positive PAs and pituitary cancer, we could not find it in our patient. Immunohistochemically, the cells showed diffuse cytoplasmic staining for cytokeratin (CAM5.2) and prolactin (**Figure 2B**). The tumor had a ki-67 index of 15% (**Figure 2C**) and increased expression of p53 (moderate in ~20% of nuclei; **Figure 2D**) but was negative for growth hormone, ACTH, TSH, LH, FSH, and GATA3. MGMT stain was not done due to non-availability. An immediate postoperative MRI showed partial resection of the central part of the huge pituitary adenoma with shrinkage of the peripheral components (**Figure 1C**). She was discharged on CBG 1 mg 4 times per week. In May 2021, she presented to the Emergency Department with severe headache and worsening visual disturbances and was found to have a large residual PA with bleeding. An MRI at the same time revealed a large invasive pituitary macroadenoma with cystic and haemorrhagic components measuring 25x36x47 mm in anteroposterior, mediolateral and craniocaudal directions (**Figure 1D**). A third TSS was deemed inappropriate and therefore, she was managed conservatively and referred to radiation oncology. She received external beam radiotherapy (XRT), 50 Gy over 25 sessions between August and September 2020. She tolerated radiotherapy well but her PA continued to grow and her symptoms got worse. An MRI in December 2021 revealed further progression of the tumor size now being 56 x 47 mm (**Figure 1E**).

**Figure 3:**
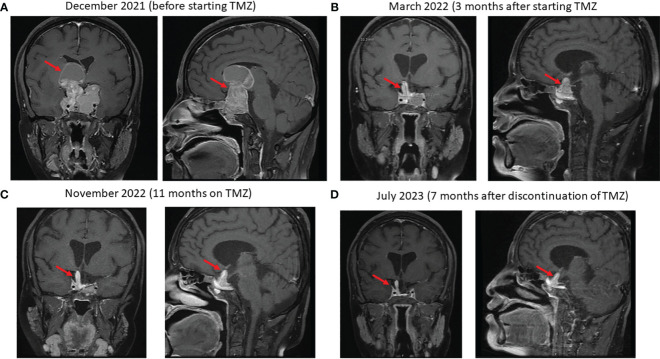
Sequential coronal and sagittal enhanced T1-weighted MR images showing the changes in the size of the macroprolactinoma (arrows) after starting temozolomide (TMZ): **(A)** at baseline just before starting TMZ, **(B)** 4 months later, **(C)** after 11 months on TMZ and **(D)** 7 months after discontinuation of TMZ.

**Figure 4:**
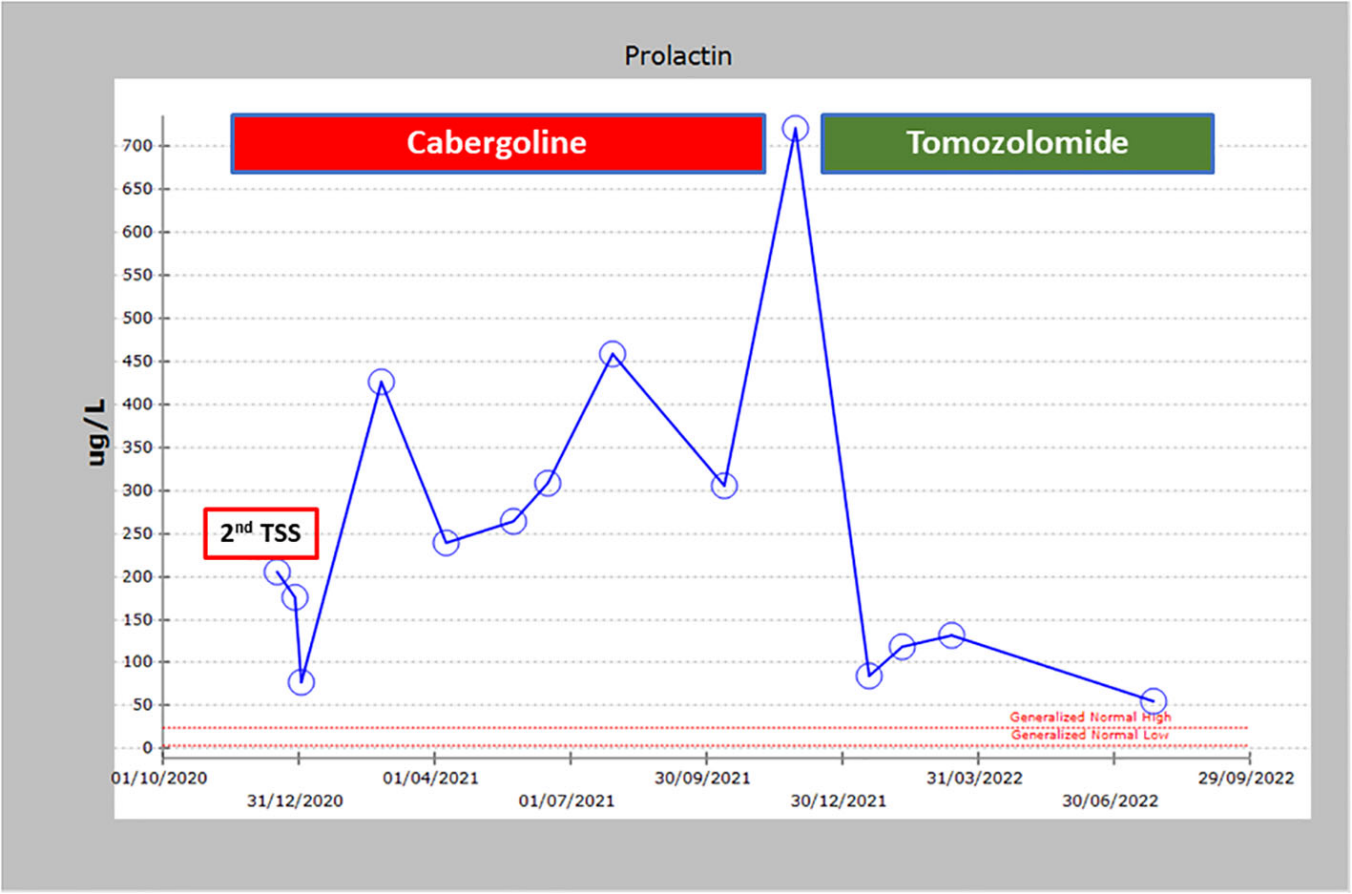
Prolactin levels over time showing the non-response to cabergoline and trans sphenoidal surgery and the dramatic response to TMZ.

Due to the large size of her resistant PA and severe symptoms, she was referred to medical oncology and was treated with temozolomide (TMZ) with dose of 300 mg daily (200 mg/m^2^) for 5 days with 3 weeks off. Her symptoms and signs significantly improved and a repeated pituitary gland MRI three and nine months later showed a remarkable reduction in the size of the PA as follows: In December 2021 (before TMZ), tumor measured 56 x 47mm (**Figure 3A**); in March 2022, measurements were 25 x 36 x 47mm (**Figure 3B**), and in November 2022, the tumor measured 21x26x33mm corresponding to 47% reduction in the size of PA to the size before TMZ treatment (**Figure 3C**). TMZ was stopped in December 2022 after a year of therapy and a repeated MRI of the PA in March 2023 and July 2023 revealed a stable size of the PA (**Figure 3D**). Prolactin level also decreased from 721 ng/ml before TMZ to 85 and 54 ng/ml in January and July 2023, respectively (**Figure 4**). The most recent laboratory evaluation (August 2023) showed prolactin level 35.2 ug/l, LH 1.9 u/l, FSH 3.0 u/l and E2 (estradiol) 27.7 pmol/l (normal follicular phase ranges 46-607). This is consistent with central hypogonadism related to PA and its management.

She is currently stable without headache. Her visual acuity and field improved but incompletely and she still has amenorrhea. Following the detection of germline *SDHB* pathogenic variant, we screened the patient for PPGL or other tumors. Plasma and 24 hr urine metanephrines, and Gallium 68 positron emission/computed tomography (Ga-68 PET/CT) were all normal.

### Molecular studies

Due to the young age of the patient and aggressive nature of PA, we performed whole exome sequencing (WES) on genomic DNA from peripheral blood. After obtaining an Institutional Review Board approval and informed consent from the patient, we isolated DNA from peripheral leucocytes using QIAamp DNA Blood Mini Kit (Cat. No. 51104, QIAGEN GmbH - Germany) and from the PA tissue using QIAamp DNA FFPE Tissue Kit (Cat. No. 56404, QIAGEN GmbH – Germany).

### Whole exome sequencing

WES of the genomic DNA was performed using the Ion Torrent platform (AmpliSeq kit). Briefly, 100 ng DNA were amplified using AmpliSeq HiFi mix (Life Technologies, Carlsbad, CA, USA) for 10 cycles. The PCR products were pooled, followed by primer digestion using FuPa reagent (Life Technologies, Carlsbad, CA, USA). This was followed by a ligation step using Ion P1 and Ion Xpress Barcode adapters. The library was purified and quantified using qPCR and the Ion Library Quantification Kit (Life Technologies, Carlsbad, CA, USA). The emulsion of the libraries was done by Ion OneTouch System to attach the DNA fragments to the Ion Sphere particles. The final step in the library preparation was the enrichment of the Ion Sphere particles using Ion OneTouch ES (Life Technologies, Carlsbad, CA, USA). Following that, the library was loaded on the sequencing chip which is then inserted into the Ion Proton instrument (Life Technologies, Carlsbad, CA, USA) for sequencing.

### Bioinformatics analysis

**Figure 5:**
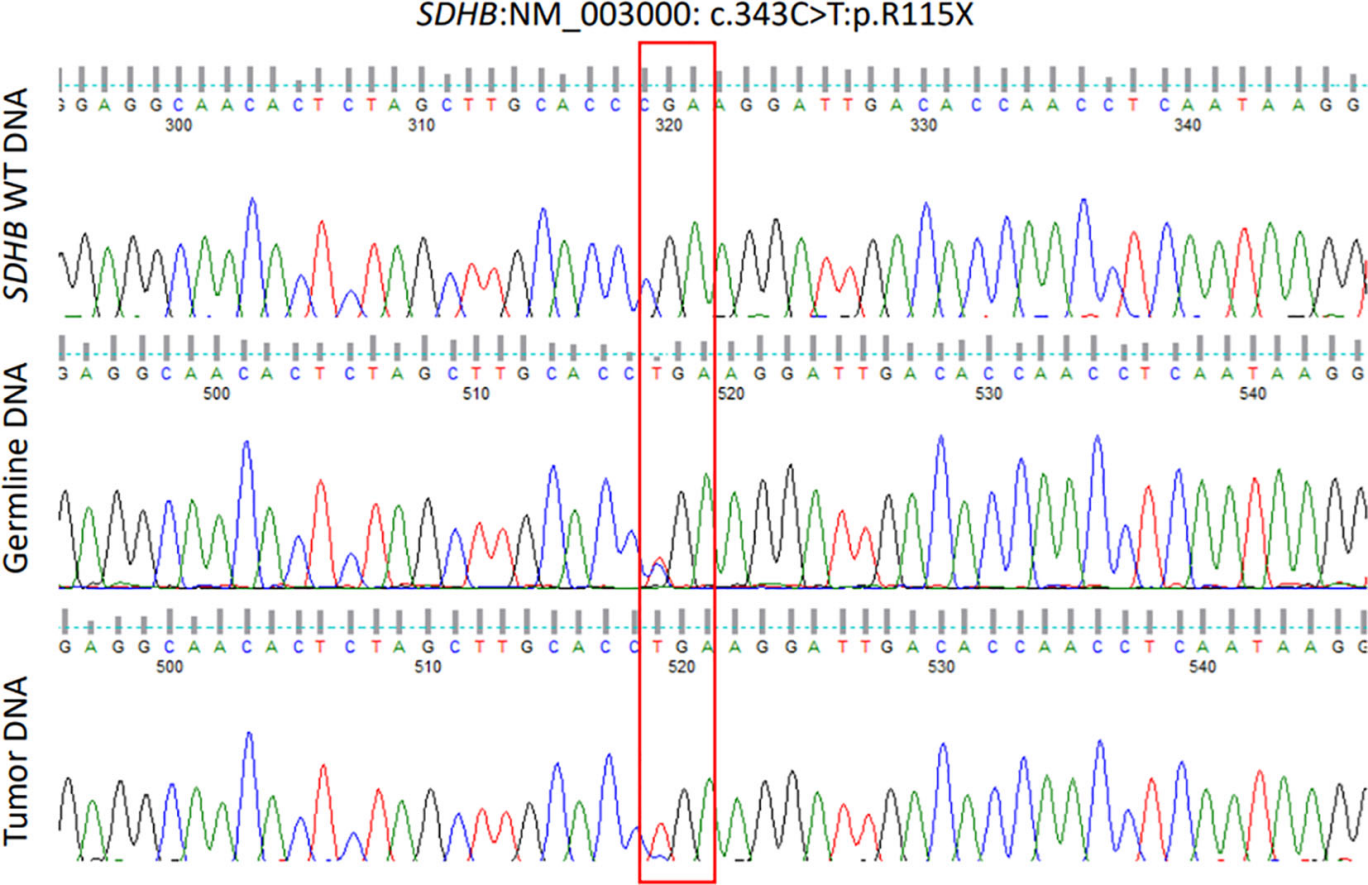
Chromatograms of part of *SDHB* exon 4 showing wild type sequence (upper panel), leucocyte DNA sequence (middle panel) showing a heterozygous germline mutation (NM_003000, c.343C>T) and pituitary adenoma (somatic) DNA sequence (lower panel) showing the same mutation in a homozygous form (loss of heterozygosity).

We removed all intronic and ncRNA variants and focused on exonic, exonic-splicing and splicing variants based on RefSeq annotation database. We also removed all synonymous variants. We followed the American College of Medical Genetics and Genomics (ACMG) criteria in assigning pathogenicity levels to the variants. We limited our analysis to genes that were reported before to be involved in the germline, mosaic, or somatic pathogenic variants of PA (*AIP, GNAS, MEN1, DICER1, CDKN1A, CDKN1B, CDKN2B, CDKN2C, PIK3CA, NF1, USP48, USP8, GPR101, PRKARIA, PRKACB, MAX, CABLES1, SF3B1, CDH23, ATRX, MLH1, MSH6, NR3C1, PMS2, TP53*, and the *SDHx* group). No pathogenic, likely pathogenic or variant of unknown significance (VUS) was found in any of these genes except *SDHB* in which we identified a previously reported pathogenic variant in *SDHB* (NM_003000:c.C343T, p.R115*). We confirmed this pathogenic variant by Sanger sequencing in forward and reverse directions (**Figure 5**) using previously published primers and PCR conditions (19). To ascertain LOH, we also performed Sanger sequencing of the same exon (exon 4) on PA tissue-derived DNA (**Figure 5**). The pathogenicity of this pathogenic variant in the PA of this patient was further confirmed by absence of SDHB staining on immunohistochemical examination (**Figure 2E**). A 15-year old paternally related niece presented with hypertension and palpitations and her evaluation (elevated urine noremetanephrines 4 times the upper limit of normal, positive Gallium 68 PET CT scan and histopathological examination of the resected PGL) confirmed the diagnosis of a para aortic upper abdominal 5 cm PGL. Examination of DNA extracted from the peripheral blood by PCR and Sanger sequencing showed the presence of same *SDHB* variant (c.343C>T). The rest of the family members are being scheduled for *SDHB* mutation testing but this testing has not been started.

## Discussion

In this report, we present a young woman with a giant macroprolactinoma resistant to large doses of CBG and rapidly growing after each of the two TSS that she underwent. Her aggressive macroprolactinoma did not respond to XRT and continued to grow despite large doses of CBG. She showed an impressive response to TMZ. The pattern and the course of this macroprolactinoma is consistent with the definition of an aggressive PA (6). The European Practice Guidelines for management of aggressive pituitary tumors recommend this diagnosis to be considered “in patients with a radiologically invasive tumour and unusually rapid tumour growth rate, or clinically relevant tumour growth despite optimal standard therapies (surgery, radiotherapy and conventional medical treatments)” (6). To understand the reasons behind this aggressive course, we performed WES and this revealed a previously described pathogenic *SDHB* variant. This was unexpected considering lack of personal and family history of *SDHx*-related tumors at the time of her presentation. Her evaluation excluded PPGL or any other related tumors. Although family history was initially negative for any *SHDx*-related tumors, a young niece has recently been diagnosed with an abdominal PGL and was found to have the same *SDHB* pathogenic variant. The absent SDHB staining on immunohistochemistry and the LOH in the tumor tissue are essentially confirmatory of the principle role of the *SDHB* pathogenic variant in the pathogenesis, aggressiveness and resistance of the macroprolactinoma in this patient.

Mutations in *SDHx* subunits are commonly associated with the development of PPGL and transmitted in an autosomal dominant pattern (13). *SDHB* mutations are particularly associated with a high risk of metastatic and aggressive PPGL (20). While mutations in *SDHx* are characteristic of PPGL, they are rarely associated with other tumors including renal cell cancer (15), gastrointestinal tumors (14) and others (17). Their association with PA has been rarely described, usually in the context of an *SDHx*-associated PPGL (sometimes referred to as the 3P syndrome) (18, 21–23). Although *SDHx* somatic variants have been described in PAs, germline pathogenic variant are extremely rare (18). These were recently reviewed by Laughrey et al. (18). Only 41 cases of *SDHx*-associated PAs have been reported in the literature including 19 *SDHB*, 9 *SDHD*, 7 *SDHA*, 5 *SDHC*, and 1 *SHDAF2* pathogenic variants (18). The most common tumor types were prolactinomas (24 cases), acromegaly (6 cases), non-functioning PA (6 cases), Cushing disease (4 cases) and PA of unclear nature (1 case) (18). Of these 41 cases, 28 (68%) were macroadenomas, 10 (24%) microadenomas and 3 (7%) of undefined size (18). Most of these cases occurred in patients with PPGL. However, only eight of them were investigated further to assess the possible role of *SDHx* variants in the pathogenesis of PA in these patients (18). The significance of the described variants in the other 33 cases of PA was not investigated and they could very well be coincidental or non-pathogenic polymorphisms (18). It is important to highlight that prolactinomas are the most common PA associated with *SDHx* mutations and since prolactinomas are rarely surgically treated, the frequency of *SDHx* variants in PA in general and prolactinomas in particular might be underestimated.

Regarding *SHDB*-associated PAs, only 19 of these 41 PAs (46%) cases have been reported so far (18). However, the pathogenic role of these variants has also rarely been confirmed (18). Of the 19 cases reported, only five (26%) underwent further tumor tissue evaluation. Three of these five cases showed LOH suggesting a role of the *SDHB* variants in the development of these PAs. The other two were negative, and in the remaining 14 cases, tumor tissue was not examined (18). Therefore, PA in these 16 cases in which an *SDHB* pathogenic variant was found might be due to *SDHB* mutations or just coincidental. In our patient, molecular and immunohistochemical examination of the PA tissue revealed LOH at the same *SDHB* codon and absent SDHB immunostaining. These essentially confirm the principle role of this pathogenic variant in the pathogenesis of PA in this patient. It also likely explains the aggressiveness and resistance of PA to CBG therapy. Therefore, our case is the fourth case of PA that has been clearly shown to be due to an *SDHB* pathogenic variant.

Our case has other interesting aspects. This is an *SDHB*-associated PA in the absence of PPGL. In the review by Laughrey et al, all cases reported in the literature had PPGL in addition to PA or family history of *SDHB*-associated PPGL (18). While our patient may develop PPGL in the future, as of now, she has been having PA for > 10 years without evidence of PPGL. It is also interesting that although formal testing for her family members has not been performed, no report of any known PPGL except in her recently diagnosed niece with PGL but without PA.

The second interesting aspect is the aggressiveness of PA and resistance to CBG and XRT, both are likely to be related to the presence of the *SDHB* pathogenic variant. On the other hand, her response to TMZ is impressive. It is interesting that another case with an *SDHB*-associated pituitary cancer also showed a similar dramatic response to TMZ (24) suggesting an underlying pathogenic mechanism that makes these *SDHB*-mediated pituitary tumors susceptible to the alkylating agent, TMZ, probably by increasing the proapoptotic or other cytotoxic effects of TMZ. Data on TMZ and factors associated with good response to it in aggressive PA or pituitary cancer remain limited and mostly based on case reports, case series or uncontrolled cohorts but still very valuable in clinical practice. In a systematic review published in 2021 and reported the follow-up of 429 patients of whom 302 had aggressive PAs and 127 had pituitary cancer, 41% showed a radiological response and 53% of those with functioning PA showed biochemical response (25). The 2-year and 4-year survival rates were 79% and 61%, respectively (25). TMZ prolonged the median progression-free survival and overall survival by 20.18 and 40.24 months, respectively (25). TMZ-related adverse events occurred in 19% of patients (25). The response to TMZ was much higher in those PA with low/intermediate O6-methylguanine (O6-MeG)-DNA methyltransferase (MGMT) expression than those with high-MGMT expression (>50%) (p < 0.001) and in functioning than in non-functioning PAs (p < 0.001) (25). Concomitant XRT significantly increased the response rate (p = 0.007) (25).

In summary, we have described a unique case of a highly invasive, aggressive and resistant giant macroprolactinoma due to a germline *SDHB* pathogenic variant in a patient without evidence of PPGL. The *SDHB* pathogenic variant most likely underpins the development, aggressiveness, and resistance of PA in this patient to CBG and XRT. Wether the *SDHB* pathogenic variant contributed to the impressive and durable response to TMZ or not remains to be investigated in future cases. This report also confirms that *SDHx* pathogenic variants might be added to the potential causes of aggressiveness and resistance of PAs to dopamine agonists that could highly benefit from TMZ treatment.

## Data availability statement

The data presented in the study are deposited in the GenBank repository, accession numbers OR817759 and OR817760.

## Ethics statement

The studies involving humans were approved by Office of Research Affairs, King Faisal Specialist Hospital & Research Centre, Riyadh, Saudi Arabia. The studies were conducted in accordance with the local legislation and institutional requirements. The participants provided their written informed consent to participate in this study. Written informed consent was obtained from the individual(s) for the publication of any potentially identifiable images or data included in this article.

## Author contributions

AA: Conceptualization, Supervision, Writing – original draft, Writing – review & editing. AN: Investigation, Methodology, Writing – original draft. MA: Investigation, Methodology, Writing – review & editing. YM: Data curation, Writing – review & editing. DP: Investigation, Writing – review & editing. HH: Investigation, Methodology, Writing – review & editing. KP: Writing – review & editing.
